# Feasibility Study on Measuring the Particulate Matter Level in the Atmosphere by Means of Yagi–Uda-Like Antennas

**DOI:** 10.3390/s20113225

**Published:** 2020-06-05

**Authors:** Aarón A. Salas-Sánchez, Julian Rauch, M. Elena López-Martín, J. Antonio Rodríguez-González, Giorgio Franceschetti, Francisco J. Ares-Pena

**Affiliations:** 1 CRETUS Institute, Department of Applied Physics, University of Santiago de Compostela, E-15782 Santiago de Compostela, Spain; aaronangel.salas@usc.es (A.A.S.-S.); rauchju93@googlemail.com (J.R.); ja.rodriguez@usc.es (J.A.R.-G.); 2 ELEDIA@UniTN, Department of Information Engineering and Computer Science, University of Trento, 38123 Trento, Italy; 3 CRETUS Institute, Department of Morphological Sciences, University of Santiago de Compostela, E-15782 Santiago de Compostela, Spain; melena.lopez.martin@usc.es; 4 Department of Electrical Engineering and Information Technology (DIETI), Università di Napoli Federico II, 80131 Naples, Italy; gfrance@unina.it

**Keywords:** air pollution, antenna arrays, dielectric constant, optimization methods, Yagi–Uda antennas

## Abstract

In this work, the application of a technique for monitoring changes of the dielectric constant of the atmosphere caused by the presence of pollution is discussed. The method is based on changes in the reflection coefficient of the device induced by these dielectric constant variations of the surrounding medium. To that end, several Yagi–Uda-like antenna designs with different size limitations were simulated by using a Method-of-Moments software and optimized by means of a simulated annealing strategy. It has been found that the larger the optimal elements of the array are allowed to be, the higher the sensitivity reached. Thus, in a trade-off between sensitivity and moderate length (regarding flexibility purposes), the most promising solution has been built. This prototype has been experimentally tested in presence of an artificial aerosol made of PAO (polyalphaolefin) oil and black carbon inclusions of a size of 0.2 μm. As a result, potentials for developing a measurement procedure by means of changes in the characteristic parameters of the antenna led by different concentration levels of suspended particles in the surrounding medium are shown. In this manner, a local mapping of polluted levels could be developed in an easy, real-time, and flexible procedure.

## 1. Introduction

Airborne particulate matter (PM) is the most generally harmful component of air pollution in European countries: around 400,000 premature deaths per year directly related with it have been estimated [1]. As known for many years, this kind of pollution affects the respiratory and/or cardiovascular systems (a study developed in 2011 concluded that it triggers almost 5% of all heart attacks [2]). Over the past decade, evidence has accumulated that PM can also affect the brain [3,4]. In addition, PM pollution was classified by the International Agency for Research on Cancer as carcinogenic [5].

To really understand the effects of PM on the Earth’s climate system and human health, it is necessary to routinely monitor PM_2.5_ (roughly, particles with an aerodynamic diameter less than 2.5 μm) on a global basis. This task is challenging, because these aerosols with less than 2.5 μm diameter particles are highly variable in space and time. Typically, the PM_2.5_ mass concentration of ambient particles is widely measured by using ground-based instruments, in both urban and rural areas of Europe, United States, Australia, and Asia. However, these ground-based observations allow just point measurements, so that the necessary coverage to map the regional to global distribution of aerosols is missing. 

As environment-related issues become increasingly important in the present and future times, developing measurement systems to quantify environmental variables also acquires greater attention.

Several overviews of the available instrumentation for measurement of particulate matter can be found in the literature [6,7,8]. More precisely, the application to scientific research purposes has been highlighted in [8]. In this review, both results regarding mass concentration and particle size have been analyzed. Regarding the objective of the present paper, the PM mass concentration techniques are the types of technology which we must be focused on. Accordingly, the classification developed in [8] about these measurement techniques includes gravimetric, optical, and microbalance methods. Gravimetric methodologies are based on weighing samples before and after the deposition of filtered particles. Optical methods are based on three principles: light scattering, light absorption, and light extinction. Additionally, for the last one, microbalance methodologies are based on changes of the resonant frequency of an oscillatory microbalance element when it is covered by a deposition of particles suspended in the environment under testing. Two of the most common microbalance technologies include tapered element oscillation microbalance (TEOM) and quartz crystal microbalance (QCM).

As gravimetric methodologies are based on the deposition of filtered particles [6,7,8], they present limitations in terms of real time response and maintenance requirements (due to a frequently replacement of filters). 

Furthermore, microbalance technologies present limitations with regard to an easy and real-time answer. To overcome such limitations (mainly in time response) a combined performance of optical methods with these technologies has been proposed [9]. Here, the use of light absorption and extinction instruments (aethalometer, photoacoustic instrument, and smoke meter) in parallel to the TEOM has been analyzed, in the particular case of measuring organic carbon particles. The differences between these methods are mainly related to the measurement parameter they are based on for detecting the masses of the particles which are deposited. Therefore, the filtering replacement drawback has also to be addressed in these alternatives.

Additionally, an interesting study that addresses the performance of one beta attenuation monitoring and six light-scattering based devices for determining PM_2.5_ mass concentrations has been developed [10]. Another example can be found in [11], wherein photoacoustic and interferometric detection methods have been discussed. Complexities in the set-up for developing a stable and flexible solution based on interferometry can be highlighted.

Regarding measurements of atmospheric particles by light absorption principle, the most relevant approaches in the state-of-the-art techniques are discussed in [12,13]. Challenges of this scenario [13] are highlighted in regard to the different cases in the description, which range from the previously referred to drawback of using filters to challenge scenarios of interferences due to light-induced particle evaporation effects, among others.

Going further, technologies based on optical methods applied to ground-based [14,15,16] and satellite-based [17,18,19] measurement systems can be highlighted. The working principle of these devices is based on measuring the aerosol optical depth (AOD). More precisely, regarding ground-based deployments, AERONET (aerosol robotic network) collaboration is extensively described [14,15]. Additionally, as an application of these approaches, comparisons between measurements of California and Nevada during the summer season of 2012 have been described [16]. On the basis of satellite applications, different reviews have been proposed [17,18,19]. Additionally, in the work developed by Donkelaar et al. [20], promising results regarding measurements of PM particle concentration have been demonstrated. However, many factors can affect the relationship between AOD and PM_2.5_. For example, the satellite-derived quantities provide columnar information for ambient conditions, whereas the particle measurements are representative of near-surface dry mass concentrations. Finally, it is worth emphasizing—from the scope of this work—that satellite footprints represent large spatial areas and are subject to cloud contamination [21].

Another interesting work, developed by Mazzoleni et al. [22], has been devoted to describing a measurement system for particulate matter emissions in an automotive scenario. These devices are based on LiDAR (light detection and ranging) methodologies, but as a drawback it is noteworthy that these systems present huge dimensions, and they are consequently not easy to manipulate. 

Other approaches have reported the use of radiofrequency antennas as measurement systems. More concretely, applications based on the measurement of gaseous pollution can be found. For instance, a complicated piece of equipment for managing the obtained data from a SODAR sensor (sonic detection and ranging) or a RASS (radio acoustic sounding system) has been described [23]. In both cases, the methodology implies the coexistence of data from three different frequencies into radio channel antenna choices. 

In the work of Tonouchi [24], a system based on a quantum cascade laser (QCL) was described. Here, the detection of hazardous gases by means of this approach has been addressed in the frame of THz frequencies.

In summary, the above-mentioned methodologies represent complex alternatives which do not allow both flexibility and real time exploration of the surrounding medium by means of easy and cheap apparatus. Additionally, they cannot map a PM polluted scenario located in a concrete region of the space-time (i.e., not on a global basis) with enough resolution to guarantee a quick analysis within a variable pollution scenario.

To fill this gap, the idea we propose here is to exploit the alterations in the relative permittivity of a medium through its changes in composition. Variations of the dielectric constant of a medium which presents PM pollution represent the central concept of this methodology. In this manner, the monitoring of concentration levels of these main actors in the atmospheric pollution scene is studied. This idea was previously discussed in a precedent work [25], where a first approach of this feasibility study was developed. In this work, the analyzed solution to measure the concentration level of PM was based on a linear array of shunt slots in the broad wall of an air-filled rectangular waveguide. However, in spite of the fact that the results shown in the work are promising, a device based on a waveguide tends to be quite complicated and expensive to manufacture.

Consequently, the approach from Yagi–Uda-like antennas seems to be a good alternative to develop this research due to their low-cost construction requirements. In addition, this type of antenna represents a robust solution for easy matching to a feeding network [26]. Therefore, this proposal consists of a linear array of dipoles [27], one of which—the so-called active element—is fed by a power source. The rest of the elements act as parasites and are excited due to near-field coupling effects induced by the driven element. Otherwise, Yagi–Uda antennas can be adopted as an advantageous alternative in front of the existing current methods because they are portable, compact, and unaffected by movement. Due to this fact, a sensor like this could be easily mounted on drones or airplanes to mapping the evolution and the dissemination of PM clouds quite easily.

It is worth mentioning that structures based on the Yagi–Uda philosophy have been also described in prior works [28] as promising solutions for monitoring relative humidity. This environmental parameter is important for the electromagnetic study of the PM inclusions. As it will be shown in next section, the dielectric constant of pure air can be expressed as a function (among other components) of the relative humidity level in the medium. It can be inferred that this effect is around one order of magnitude less intense than that produced by an average concentration of PM in conditions reported in the literature (see Supplementary Materials of this paper for more information). Despite this fact, it will be necessary to know its value to adjust our measurement system. In this case, the proposed device was based on a Yagi–Uda-like antenna whose elements were coated with a thin layer of polyimide. This coating was crucial to the good performance of the antenna due to its hygroscopic properties. This study was focused on the evaluation of changes in the antenna by means of its resonant frequency which are led by the interaction of the water content in the air with the polyimide compound.

Therefore, in this work, a solution regarding the PM concentration level measuring problem at the atmospheric level in terms of Yagi–Uda-like antennas is developed. This purpose is motivated by the necessity of providing a flexible, economical, and efficient alternative to the existing measurement methods. Some promising results about this methodology are illustrated in this paper.

## 2. Materials and Methods

### 2.1. Background Theory

#### 2.1.1. Main Antenna Parameters

It is well known that the voltage standing-wave ratio (VSWR) of an antenna describes the level of impedance matching to a transmission line. This quantity, as defined in the literature [29,30], is given by

(1) $$VSWR=\frac{\left|A|+|B\right|}{\left|A|-|B\right|},$$

where |A| and |B| denote the amplitude of the incident and backscattered waves in a reference point of a port, respectively. 

Alternatively, the voltage reflection coefficient (also known as S_11_ parameter for a network with just one port), in the case of a connection to a load ($Z_{L}$), is expressed as

(2) $$S_{11}=\Gamma =\frac{B}{A}=\frac{(Z_{L}/Z_{0})-1}{(Z_{L}/Z_{0})+1},$$

leading to

(3) $$VSWR=\frac{1+\rho }{1-\rho },$$

where ρ=|Γ|, and $Z_{L}$, $Z_{0}$ are the load impedance and the characteristic impedance of the line respectively. 

Consequently,

(4) $$\rho =\frac{VSWR-1}{VSWR+1}$$

can be obtained as the definition of the reflection coefficient (ρ) of an antenna in terms of the *VSWR*. If the antenna is matched to a specific frequency $f_{0}$ and relative permittivity $\varepsilon_{r}$, i.e., $Z_{L}=Z_{0}$, the reflection coefficient defined in Equation (2) is equal to zero.

Changes in the surrounding medium, namely, its relative permittivity $\varepsilon_{r}$, will lead to a different wavelength due to the relation

(5) $$\lambda =\lambda_{0}/\sqrt{\varepsilon_{r}},$$

where $\lambda_{0}$ is the wavelength corresponding to $\varepsilon_{r}=1.00$.

Thus, in the basis of this model, adding pollution to pure air $\left(\varepsilon_{r}≃1.00\right)$ is translated into changes of the dielectric constant in the medium into Equation (5). In other words, it can be concluded that the wavelength will vary and the antenna is no longer matched. This represents the effect from which this measurement system is exploiting. The changes in terms of dielectric constant will be referred to the real part of this parameter.

#### 2.1.2. Relative Permittivity of Air in Presence of PM

The relative permittivity of air is often assumed to be almost a unit, but it is in fact a function of temperature *T* in Kelvin, air pressure *P* in millibars, and the water vapor pressure *V* in millibars,

(6) $$\varepsilon_{r}={\left[1+10^{-6}\left(\frac{79P}{T}-\frac{11V}{T}+\frac{3.8\times 10^{5}V}{T^{2}}\right)\right]}^{2},$$

as shown in the literature [29,31]. Additionally, a more detailed analysis could be realized from this formula by checking the more accurate range of values which can be produced by assuming a real atmospheric environment. The limits of this model are the following [31]:

- Temperatures of −50 °C to +40 °C;
- Pressures of 200 mbar to 1100 mbar;
- Water vapor partial pressures up to 30 mbar;
- Frequency range up to 30 GHz.

All these restrictions are translated into a variable limitation on the accuracy for the relative humidity, directly linked with the temperature level. For instance, numerical analysis was performed in order to check the validity of this formula, and it can be concluded that, for average conditions on Earth, the most extremely high value reached of this dielectric constant is not more than 1.0008 (a detailed information can be found in the Supplementary Materials of this paper, and a mathematical definition of the semi-empirical formulation of the water vapor pressure). Otherwise, by assuming a pressure of 1 atm (i.e., 1013.25 mbar), a relative humidity level of 60%, and a temperature of 20 °C, the relative permittivity of pure air is about 1.00067. This value will be used in this work to evaluate the performance of our method in terms of concentration level of PM particles.

Using spherical inclusions with permittivity $\varepsilon_{i}$, which occupy a volume fraction f (volumetric fraction of the inclusions to the total volume of the mixture) within a host material with relative permittivity $\varepsilon_{e}$, the resulting effective relative permittivity $\varepsilon_{eff}$ can be calculated [32]:

(7) $$\frac{\varepsilon_{eff}-\varepsilon_{e}}{\varepsilon_{eff}+2\varepsilon_{e}+\nu (\varepsilon_{eff}-\varepsilon_{e})}=f\frac{\varepsilon_{i}-\varepsilon_{e}}{\varepsilon +2\varepsilon +\nu (\varepsilon_{eff}-\varepsilon_{e})}.$$

By varying the dimensionless parameter *ν*, the Maxwell–Garnett rule (*ν* = 0), the Bruggeman formula (*ν* = 2), and the coherent potential approximation (*ν* = 3) are considered. A summary of the parameters involved in the effective medium theory description through Equation (7) is reflected in Table 1. The real part of the relative permittivity has been always considered.

#### 2.1.3. Input Impedance of a Yagi–Uda-like Antenna

**Figure 1:**
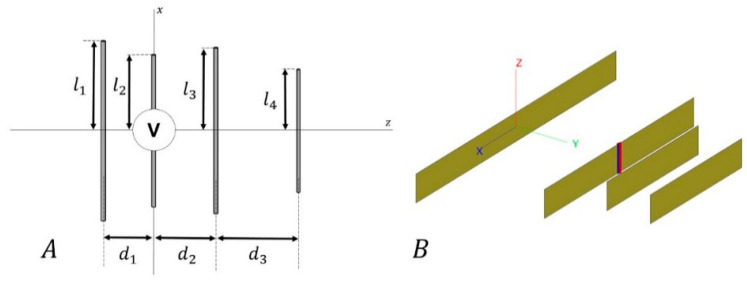
(**A**) Sketch of a Yagi–Uda antenna. The length of one dipole i is twice the length $l_{i}$. The distance $d_{i}$ between the dipoles denotes spacing between the element i and i+1. All these variables are expressed in terms of λ. The second element was used as the active element. (**B**) Detail of a Yagi–Uda antenna model in the 3D electromagnetic simulation software suite FEKO [33].

According to the proposed configuration (Figure 1A), and since the active dipole is the second element, the input impedance is given by [27]:

(8) $$Z_{in}=Z_{22}+\sum_{i=1,i\neq 2}^{4}\frac{I_{i}}{I_{2}}Z_{2i}=Z_{22}+Z_{2}^{C},$$

where $I_{i}$ denotes the current in the element i, $Z_{22}$ the self-impedance of the active element, and $Z_{2i}$ the mutual impedance (i≠2) due to electromagnetic coupling between the elements. We shall loosely refer to $Z_{2}^{C}$ as the mutual coupling term, or more simply, the mutual coupling. As it is shown in the literature [27], the input impedance depends on the values of $l_{i}$ and $d_{i}$ (see Figure 1A).

### 2.2. Numerical Simulation Settings

#### 2.2.1. Antenna Model and Environmental Scenario

As aforementioned, the array antennas designed in this paper are based on the Yagi–Uda array structure. A sketch of this type of structure and the model implemented can be seen in Figure 1. In this concrete case, the Yagi–Uda-like antennas are based on 4 elements (1 reflector, 1 active, and 2 directors). The main reason to choose this number of elements is to guarantee a small boom length and to promote an inexpensive device. Solutions with even smaller numbers of elements (2 or 3) were also tried in the present work, but without yielding promising results.

The main parameter of the antenna to analyze the potentials of this methodology for measuring the PM concentration level is the reflection coefficient. All the antennas were immersed in a simulated polluted scenario to determine its radiation behavior versus changes of the surrounding medium through its effective permittivity. Consequently, from a theoretical point of view, we understand these immersions as effective electrical length changes of the radiating elements, led by Equation (5). This will affect the effective electrical length, distances, and radii in the array antenna models.

#### 2.2.2. Optimization Strategy

**Figure 2:**
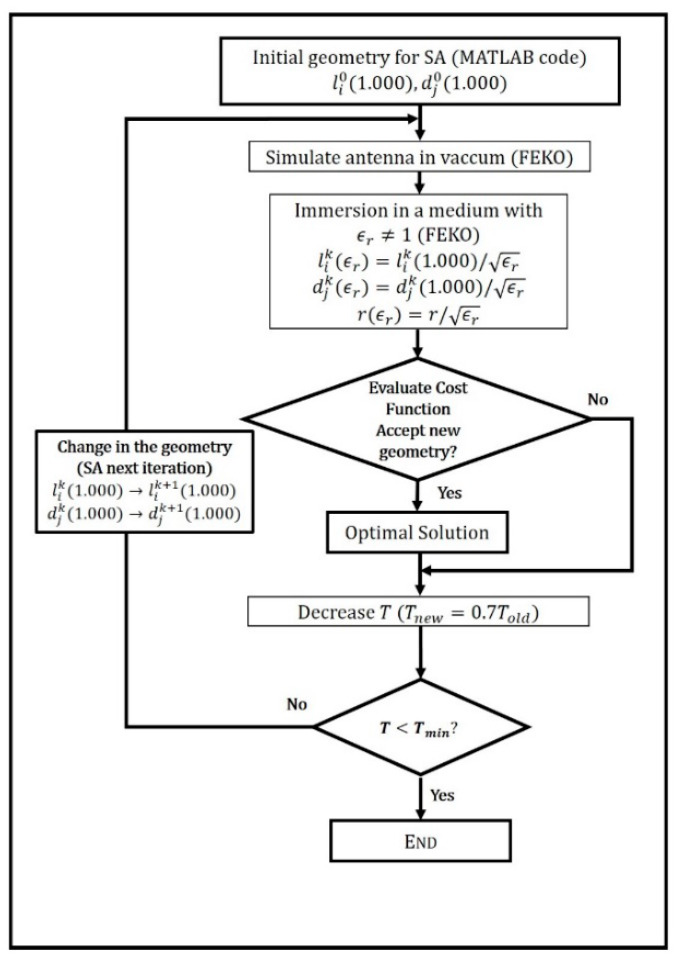
Flowchart of the optimization strategy combined with the numerical approach for the immersion process of the Yagi–Uda-like antennas in a dielectric medium. The parameters $l_{i}$ and $d_{i}$ are the lengths and spacings (in terms of *λ*) and *r* are the radii of the antennas, which have been fixed to a value of 0.75 mm in the vacuum. The initial value of the *T* parameter involved in this SA strategy (linked to the randomness of the search of the algorithm) was set as 100.

Pursuing the objective of being well matched to a transmission line, all the antennas here described were designed to match the transmission line by using a common strategy. This strategy comprises numerical simulations in FEKO software suite [33] integrated within an optimization procedure coded in MATLAB [34]. The optimization is based on a hybrid approach of the simulated annealing (SA) algorithm [35], modified by means of the downhill simplex (DS) method [36] (by following a Numerical Recipes [37] version of the code). The accuracy of each numerical analysis is guaranteed by the capabilities of FEKO as a frequency domain full-wave software based on method-of-moments (MoM) [33]. The optimization procedure varies the values of $l_{i}$ and $d_{i}$ in order to match a 50 Ω feed line to the antenna array and to improve the sensitivity of the proposed system. Therefore, a cost function to guarantee both impedance matching and sensitivity to permittivity variations was defined as:

(9) $$C=c_{1}\times \left|\rho (1.00)\right|+c_{2}\times \frac{1}{{\left|\rho (1.01)-\rho (1.00)\right|}^{2}},$$

where *ρ* is the reflection coefficient, and it depends on the value of the relative permittivity $\left(\varepsilon_{r}\right)$. Therefore, ρ(1.00) is the reflection coefficient in the vacuum and ρ(1.01) is the coefficient in a surrounding medium with a dielectric constant which differs a 1% from the vacuum case. In this sense, the sensitivity of the device against small deviations in the dielectric constant of the medium in which it is immersed is improved; $c_{1}$ and $c_{2}$ are the appropriate weights to the terms of the cost function (more concretely, in all these cases, they were $c_{1}=1$, $c_{2}=1000$). A flowchart of this optimization procedure can be seen in Figure 2, where a description of the implemented model for the immersion process of the antennas is also depicted. It is worth highlighting that for trying to look for a solution with moderate lengths, a constrained optimization was performed by setting different allowed length ranges for each element of the array. 

### 2.3. Experimental Setup

**Figure 3:**
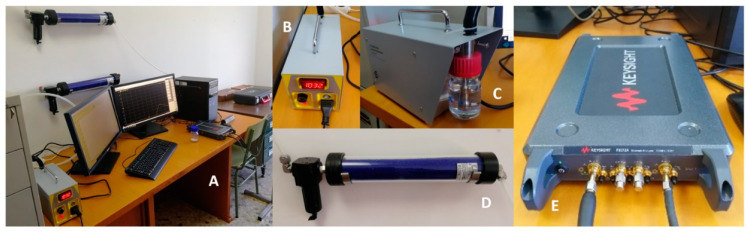
(**A**) General view of the exposure set-up. (**B**) Polydisperse test aerosol generator TSI 3073. (**C**) Detail of the liquid solution to be introduced into the system for creating the suspension of particles. (**D**) Diffusion dryer (TSI 3062) for drying the initial liquid solution introduced in (**C**,**E**) Vector Network Analyzer Keysight P9372A (300 kHz to 9 GHz).

**Figure 4:**
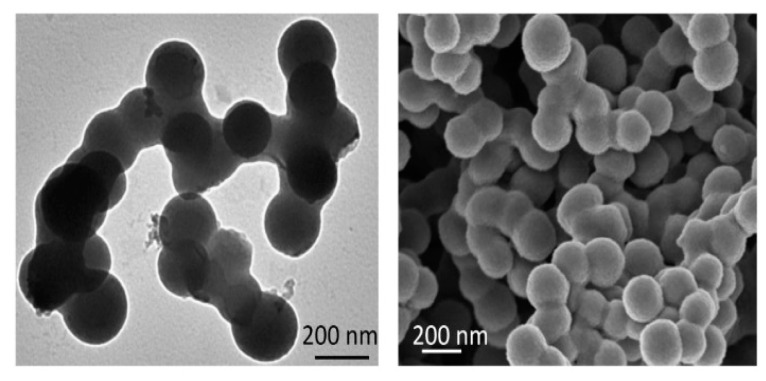
Detailed view of the black carbon samples used for a controlled exposure: Transmission (**left**) and scanning (**right**) electron microscope image.

A graphical description of the system for the exposure can be found in Figure 3. The system of exposure is based on the use of a polydisperse aerosol generator with a constant particle flow connected to a measurement box where both the antenna and the environment were controlled. The tests were conducted by generating submicron aerosols (through a generation system which can manage particle sizes from 0.01 to 2.00 μm) from a liquid suspension of black carbon [38,39] particles with a diameter of 0.2 μm (see Figure 4) into PAO (polyalphaolefin) oil. To produce a controlled environment for the exposure to the PM particles, the antenna has been introduced into a mechanically isolated cardboard box, with a size of 40 cm × 30 cm × 30 cm. A solution made with 1 g of black carbon particles in 60 mL of PAO oil has been used as input of the system (see Figure 3C) in order to create the gaseous suspension of pollutants. In addition, the maximum output air flow of 4.5 L/min, led by adjusting the maximum nozzle pressure (1032 hPa) of the polydisperse aerosol generator, has been selected. In these conditions, the polydisperse aerosol generator produces particle concentrations from 10^2^ part/cm^3^ to 10^7^ part/cm^3^ [40]. To prevent experimental artifacts and guarantee the reproducibility of the results, the vector network analyzer (Figure 3E) has been used and calibrated. 

**Figure 5:**
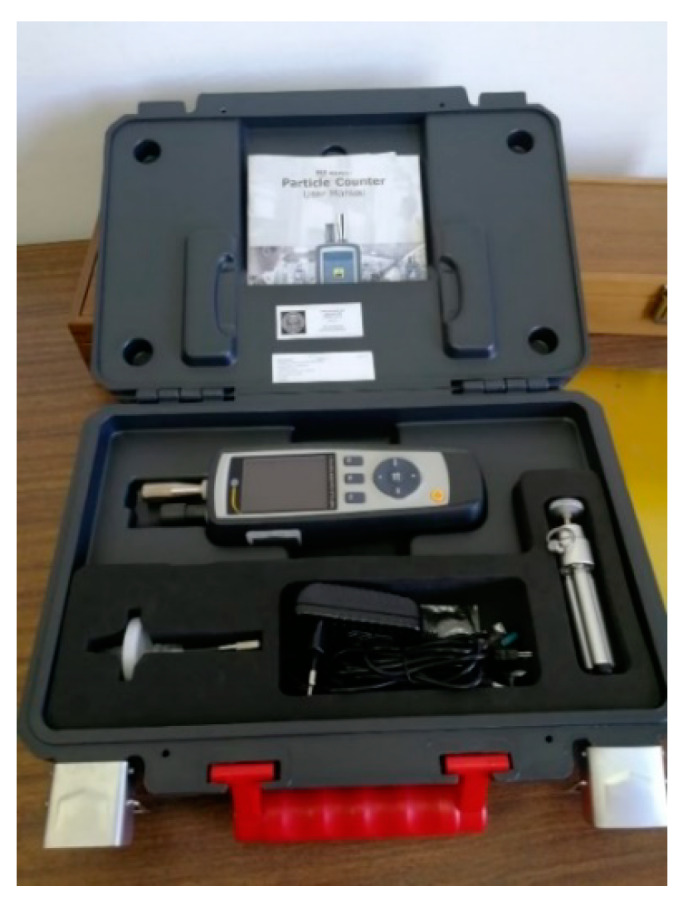
Detail of the particle counter PCE-PCO1 manufactured by PCE instruments.

At the same time, a particle counter PCE-PCO1 (see Figure 5) from PCE instruments has been used to characterize the particle distribution size generated by the system.

## 3. Results

### 3.1. Numerical Results

The resulting six most relevant antenna arrays obtained by this method are shown in Table 2. Each one of them is defined by the different lengths and spacings of its elements. The differences which can be highlighted in terms of lengths among the obtained solutions have been motivated by selecting different limits to the size of each antenna. These sizes have been obtained by restricting each antenna length to different intervals—in terms of wavelength—in order to compare performances and look for a trade-off between accuracy and flexibility. Otherwise, the limits on the spacings have not been changed.

**Figure 6:**
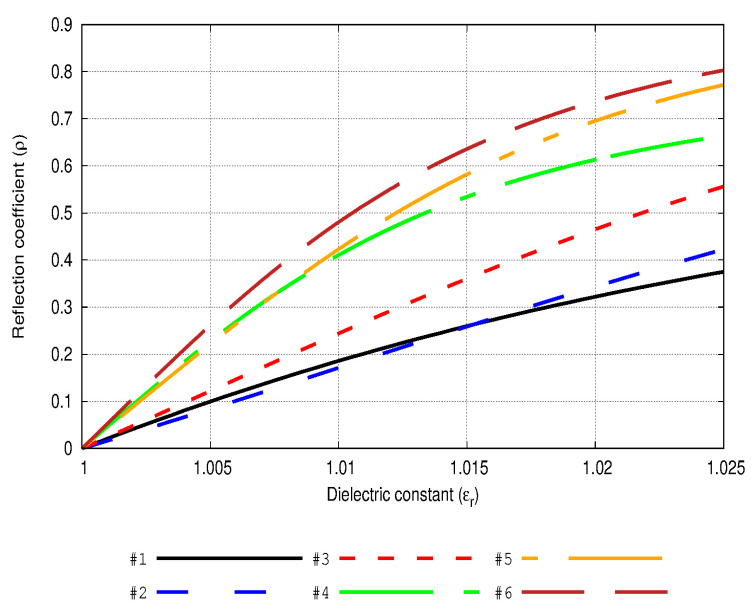
Behavior of the reflection coefficient for each simulated antenna as the enclosing medium is changed. The line number corresponds to the antenna number of Table 2. Antenna #6 shows the highest slope and reaches a value of 0.47 for the reflection coefficient when $\varepsilon_{r}=1.01$.

As a first approach, Figure 6 displays the results in terms of response of the reflection coefficient to changes in the dielectric constant of the medium in which the antennas of Table 2 are immersed. Here it can be noted that the sensitivity will vary corresponding to the size of the chosen antenna. More concretely, Figure 6 shows that small changes at low levels of permittivity values could be registered with more accuracy by using the proposed antennas but of greater size. However, if the measurement process needs to deal with higher values of permittivity, these values could be monitored through some antennas much smaller in size, such as 1, 2, or 3 (see Table 2). An interesting idea in terms of a practical solution for a global measurement system may be the development of an antenna array formed by modular elements. Therefore, different lengths can be implemented by a common device, and in this way, a more precise performance on different ranges of permittivity values could be promoted.

**Figure 7:**
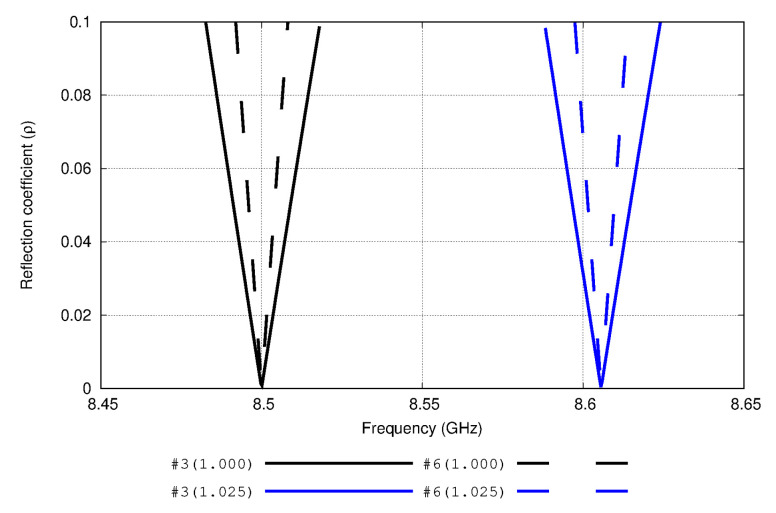
Reflection coefficients of antennas 3 and 6 (Table 2) at different frequencies in different media ($\varepsilon_{r}=1.000$ and $\varepsilon_{r}=1.025$ respectively). The resonant frequency is shifted in 106 MHz for both cases. This shift is independent of the selection of the antenna.

Figure 7 shows the behavior of the reflection coefficient of two antenna examples of Table 2: antenna number 3 and antenna number 6. By analyzing this figure, a decrease of the bandwidth is caused by the high *Q* value (the ratio between the resonance frequency and the frequency range in which the reflection coefficient is below 0.2) of the antenna number 6 versus the antenna number 3. Differences between the slopes in the reflection coefficient function are quite significant between these two examples. Otherwise, it can be interestingly noted that the frequency shift suffered by the antennas must be related with the introduction of different permittivity values. It has been found that this shift is exactly the same among all the proposed antennas of this study. This comparison is illustrated in Figure 7.

Table 3 highlights the quality factor *Q*, $Z_{22}$, $Z_{in}$, and the mutual coupling term of each antenna. Changes in complex self-impedances and mutual coupling terms based on changes in the electrical lengths of the dipoles are reflected in this table. Through these results, the mismatching effect suffered by the antennas is depicted. Although a monotonic behavior is not shown (there does not appear to be a general relation between *Q* value and impedance mismatching for all the antennas), it is clear from these values (e.g., Figure 6 and Figure 7) that a larger antenna is necessary to have a sufficient quality factor to offer good performance in terms of sensitivity to dielectric constant variations.

**Figure 8:**
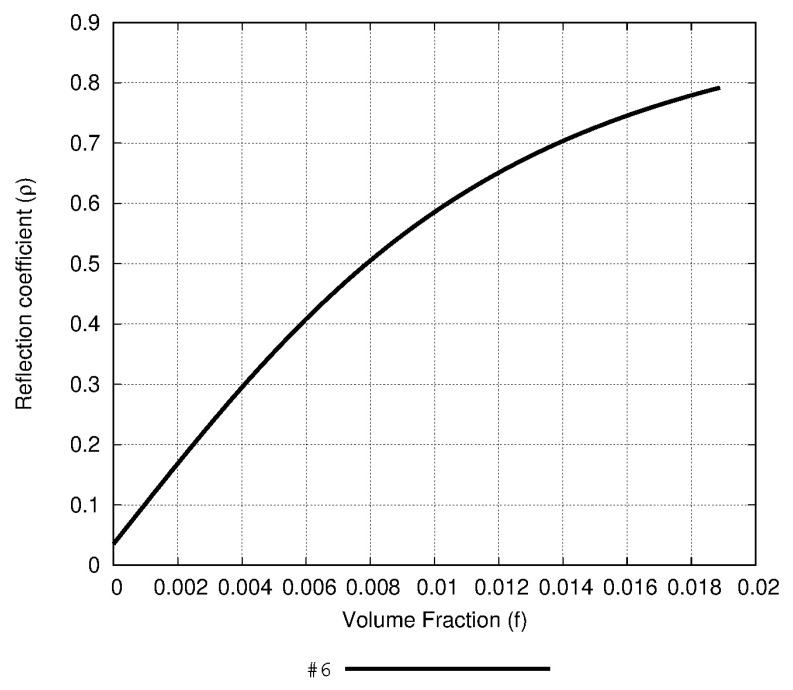
Volume fraction of a pollutant with $\varepsilon_{i}≃3.2$ and a host medium with $\varepsilon_{e}≃1.00067$ derived from the simulated reflection coefficient of antenna 6 (Table 2). Assumptions related with the Bruggeman formula have been done for the calculations.

Figure 8 shows the direct relation between the reflection coefficient and the volume fraction of the antenna 6 of Table 2 in a concrete PM inclusion (soot particles) scenario. This test case is based on inclusions of 0.2 μm of diameter (PM_2.5_), i.e., with a relative permittivity of $\varepsilon_{i}≃3.2$ [41] which is within an environment at a pressure of 1 atm, while a relative humidity level of 60% and a temperature of 20 °C were assumed $\left(\varepsilon_{e}≃1.00067\right)$. The curve was obtained by following the Bruggeman formula (setting ν=2 in Equation (7)), which assumes that the pollutants tend to be clustered [32]. Due to the smoothness of this function, it is possible to identify the reflection coefficient in terms of the volume fraction of soot particles inclusions. Therefore, by analyzing Figure 8 it can be claimed that this antenna example represents a feasible method to monitor PM polluted scenarios.

In order to discuss the feasibility of the solution here proposed, a numerical approach is suggested and implemented in the following lines. This test is based on the assumptions and parameters referred to in Section 2.1.2 and evaluating some real environmental scenarios obtained by the literature. Firstly, a concrete case of black carbon inclusions and their mixture in the air, reflected on its volume factor value is reported in Koven et al. [42]. If calculations are developed according to Equation (7), it leads to an effective relative permittivity of $\varepsilon_{r}=\varepsilon_{eff}=1.009$. The black carbon particles analyzed in that work represent a primary component in ambient pollution and diesel exhaust, and they are found in many different environmental scenarios. Thus, by focusing on a situation described in terms of the reported permittivity, the antenna number 6 of Table 2 offers the value of the reflection coefficient ρ=0.4354, and describes the curve 6 in Figure 6 with a minimum slope of 36.57 in a range $\varepsilon_{r}\in \left[1.00,1.01\right]$. This result improves the one obtained in a prior work [25], where the minimum slope of 20.88 was obtained. Additionally, from another example in the literature developed by Michel et al. [41] a scenario was created in which the resulting effective permittivity can be obtained by means of the effective medium theory model (Equation (7)). As a result, in this case the effective dielectric constant is $\varepsilon_{r}=\varepsilon_{eff}=1.023$. Particularly, the sixth antenna of Table 2 presents for this dielectric constant a reflection coefficient of ρ=0.7690.

### 3.2. Prototyping and Experimental Results

**Figure 9:**
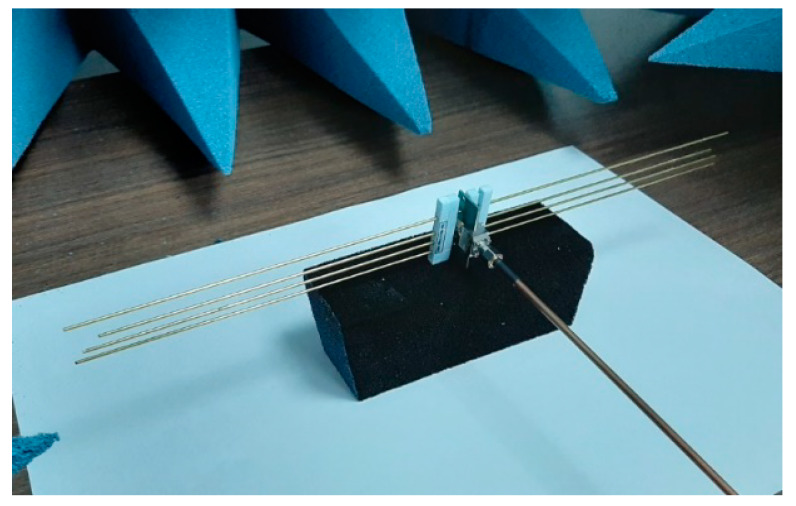
Antenna prototype based on the characteristics of the antenna solution #6 (Table 2) and built following the geometrical descriptions of Table 4 used as a proof-of-concept device for testing the hypothesis of this work.

**Figure 10:**
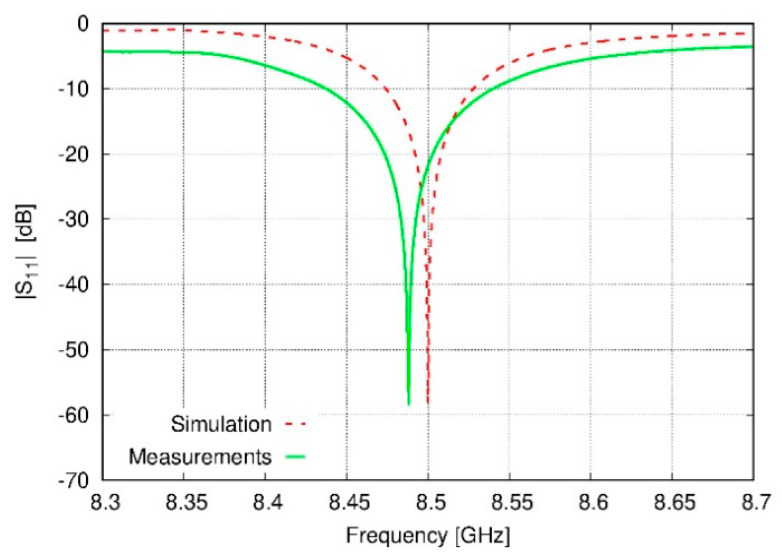
Comparison of S_11_ curves from simulated and experimental data—the latter by using the antenna designed on specifications for solution #6 of Table 2.

Based on the previous simulations, a prototype with the specifications of antenna 6 has been constructed (see Figure 9); the real dimensions are shown in Table 4. In the final design, a balun feed—based on a microstrip with optimized characteristics for matching the antenna to the line—has been introduced. A comparison between the simulated and the measured S_11_ is shown in Figure 10. Although there is an evident degree of similarity between the two scenarios, differences in terms of resonant frequency (8.488 GHz versus 8.500 GHz) and bandwidth (1.23% versus 0.63%) are reflected in the results. The inclusion of Teflon sticks—to keep the distances between the elements of the array—in addition to the use of a microstrip balun for feeding purposes are error sources which must be taken into account in order to explain these discrepancies between the data. Additionally, it must be noted that ambient parameters (as they have been discussed in the Materials and Methods section) have been playing a role in these measurements (due to the fact that the design of these devices has been developed in vacuum, instead of air with certain temperature, humidity, and pressure).

As introduced in the Materials and Methods section, a particle counter PCE-PCO1 (see Figure 5) from PCE instruments has been used to characterize the particle distribution size generated by the experimental system (see Figure 3). A constant ratio of size distribution of suspended particle concentration has been obtained by performing several measurements at different times of exposition. In this manner, the environment of suspended particles created by this set-up is of an 80% made of particles with less than 0.3 μm of aerodynamic diameter, while the other 20% is essentially filled with particles of a size between 0.3 and 0.5 μm of aerodynamic diameter. Greater particle sizes (up to the limit of the instrumentation: 2.0 μm) have a minimal impact. This is in line with the expectations, since the process of emission is implemented through a liquid suspension of the particles and by means of the diffusion dryers of the polydisperse aerosol generator. In that sense, the system could be viewed as an atomizer and is described in Section 2.3. It is worth mentioning that reviews in the literature claim that black carbon particle compounds usually are present in terms of aggregates [38,39]. Here, particles with a constant primary size of 0.2 μm (Figure 4) have been set as input and they were linked to a suspension with the PAO oil.

**Figure 11:**
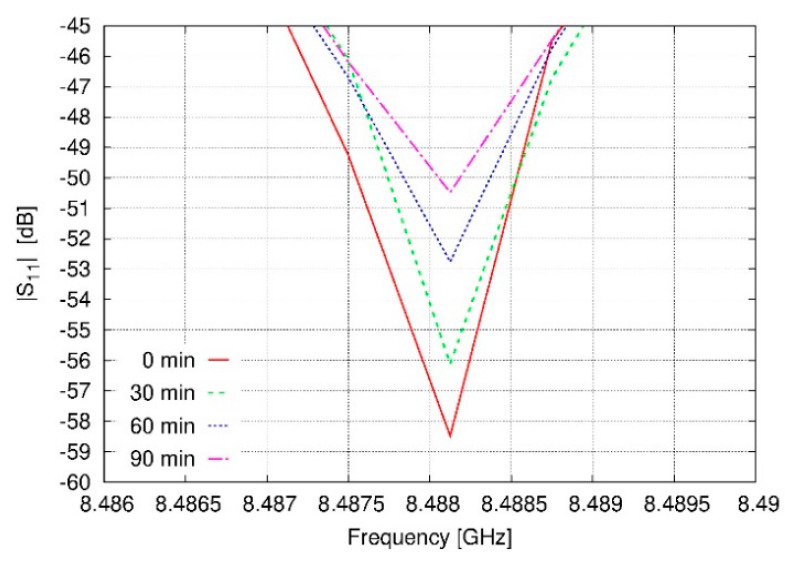
Measured |S_11_| curves of the antenna near to the resonant frequency for different times of exposure.

For evaluating the magnitude of the effects of immersing the prototype into a polluted environment, several S_11_ measurements were conducted by exposing the antenna to a different number of suspended particle concentrations (Figure 11 and Table 5). This exposition was analyzed by registering data each 10 min up to complete a series of measurements over a total time of 90 min.

With time, the exposure set-up fills the box with particles suspended in the air, and therefore, the S_11_ of the antenna is changing its value. More concretely, in order to confirm these effects, changes in |S_11_| values at the frequency of the minimum |S_11_| at initial conditions of exposure are shown in Figure 11. These changes, after 90 min of exposure to a constant flow of particles, are of 8.01 dB. In Table 5, additional time steps of this response in |S_11_| are shown. The measurement conditions were monitored, and the environment presented a temperature of 20.6 °C, a relative humidity of 62.6%, and a pressure of 1013.25 mbar during the experiment. With regard to these results and comparing Figure 7 and Figure 11, we can observe that no large shifts of the resonant frequency are appreciable. Thus, in this manner, we can judge the impact of the PM concentration level in terms of the dielectric constant of the surrounding medium was not relevant. Next steps in this research have to be focused on define experimentally the relation between the concentration level of PM particles and the dielectric constant of the air.

## 4. Discussion

In this work a study on the behavior of the reflection coefficient for different antenna solutions based on the Yagi–Uda-like structure was performed. Different antenna responses which can be related to different ranges of effective relative permittivity variations were illustrated. The mismatching process of these antennas has established the basis of an interesting solution for monitoring PM levels through electromagnetic terms. This methodology was numerically tested in reported real polluted scenarios by modelling the volume fraction of the PM inclusions with help of the effective medium theory approach. This procedure represents an innovative advance in front of recent published works in the literature. Especially, results of the proposed antenna number 6 make this Yagi–Uda-like structure a good candidate for measuring different PM levels by means of its reflection coefficient or voltage standing wave ratio (VSWR). With the aim of confirming the effects of a polluted scenario over the devices, some measurements in a controlled environment were conducted. The results are promising and a non-negligible variation in terms of S_11_ parameter has been observed. Otherwise, due to the reported small shifts in resonant frequency, future research for addressing the concrete practical sensitivity of this alternative becomes mandatory. It is worth highlighting that the exposure system described and used here during the experimental part behaves in practice as a monodisperse aerosol generator, and hence, the impact in terms of dielectric constant and concentration was limited. In this manner, also a more precise experimental setup (involving more complex exposure situations), and a characterization of the entire environment in terms of the dielectric constant are mandatory. Up to this point and following the results of this research, the idea of introducing a measurement system based on the determination of the voltage standing wave ratio (Equation (3)) is proposed here. In this manner, a cheap and “easy-to-use” technology directly linked with the measurement of the effects is reported. Thus, on this basis, a flexible and real-time exploration can be exploited.

Regarding the limitations of this methodology, it is important to point out that the reported changes on the dielectric constant of a real environmental scenario without the presence of pollutants, based only on environmental parameters temperature, pressure, and relative humidity, are one order of magnitude less than the homologous effects based on the pollutants inclusions modeled by the effective medium theory. Therefore, it is not expected to have effects from temperature, pressure, or humidity variables which could dissemble relevant measurements in PM concentration level. Despite this fact, it is mandatory to know these environmental parameters in order to improve the accuracy of the method. On this basis, parallel measurements of the environmental parameters temperature, pressure, and relative humidity have to be developed within the measurement process herein envisaged.

Otherwise, it can be concluded that a high value of Q is directly related with the fact that the antenna would act more as a resonator structure than as a radiating one. It has been proven in the literature that this behavior is highly interesting for a solution to measure the atmospheric dielectric constant in a terrestrial scenario. Additionally, it could be used for applications such as monitoring “extreme environments” (volcanic eruptions), industrial pollution, or even for extraterrestrial atmospheres. In this sense, it has been found that larger antennas offer better performance for low dielectric constant levels, but larger values of permittivity could be properly monitored with an antenna whose elements are of reduced lengths. Thus, a system which involves a modular array structure by changing its element lengths could be a solution for a more complex scenarios without a priori restricted values in the dielectric constant as the above referred ones.

Alternatively, reinforcing the interest of this methodology, a secondary advantage to exploit could be the mapping of the dielectric constant in this environmental scenario. This topic is also critical from the wireless communication technologies point of view. Changes in the dielectric constant of the atmosphere at different heights could provoke an important refraction of the electromagnetic fields which are propagating in the medium, and could possibly provide a fading phenomenon. This effect is based on the expected attenuation and change of the propagating direction of the wave, and could be sufficient to affect the wireless communication systems during the period of time when the changes of the abovementioned dielectric constant are relevant. This is based on the low height of a cloud of PM suspended in the air. Therefore, through a theoretical point of view, it is plausible that this strong change in the concentration level of PM could produce a high vertical gradient of the dielectric constant of the air. Additionally, by the analysis of this gradient, another complementary and interesting scenario in which this solution could be useful is facing temperature inversion effects in the atmosphere. In this scenario, the so-called super-refraction will occur due to the increasing of the temperature with the height and is responsible for the creation of some clouds by moving air masses and even affecting PM formations by trapping the suspended particles into a layer at stratospheric level and producing a long-term impact in climate change. Finally, it is important to note that this super-refraction effect could induce high electromagnetic field levels in the surface, and therefore, increase the electromagnetic pollution in the ambient.

Future trends for the applicability of this technology can be focused on studies about the biological effects of electromagnetic fields in dependency with the action of other agents, in some cases in a timing-dependent fashion. Accordingly, it would be necessary to investigate the above working hypothesis in a laboratory setting, to study the joint effects on animals simultaneously exposed to airborne PM and non-ionizing electromagnetic radiation, as it is proposed in [43]. In this manner, possibilities about synergy, antagonism, or facilitation between electromagnetic fields and these pollution agents can be discussed.

## Figures and Tables

**Table 1:** Description of the parameters in the effective medium theory model (Equation (7))

Parameter	Description	Units
$\varepsilon_{i}$	Relative permittivity of the inclusions	Dimensionless
f	Volume fraction of the inclusions	Dimensionless
$\varepsilon_{e}$	Relative permittivity of the host material	Dimensionless
*ν*	Control parameter for changing the model (MG, BG, CPA) *	Dimensionless
$\varepsilon_{eff}$	Resulting effective relative permittivity of the mixture	Dimensionless

* MG: Maxwell–Garnett rule; BG: Bruggeman formula; CPA: coherent potential approximation.

**Table 2:** Lengths and spacings of the relevant antennas based on the Yagi–Uda structure (described in Figure 1A) with four elements.

Antenna	Elem i	Lengths $l_{i}(\lambda )$	Spacing $d_{i}(\lambda )$	Antenna	Elem i	Lengths $l_{i}(\lambda )$	Spacing $d_{i}(\lambda )$
#1 [0.1–0.5] *	1	0.4732	0.3414	#4 [2.5–4.0] *	1	3.1841	0.2895
	2 ^a^	0.3525	0.1108		2 ^a^	3.1646	0.3277
	3	0.2157	0.1435		3	3.3171	0.2652
	4	0.4768	-		4	3.2444	-
#2 [0.5–2.0] *	1	1.2267	0.3109	#5 [3.5–5.0] *	1	4.2677	0.2303
	2 ^a^	1.1679	0.2663		2 ^a^	4.1525	0.2103
	3	1.2770	0.2722		3	4.3593	0.2568
	4	1.2867	-		4	4.1907	-
#3 [1.5–3.0] *	1	2.2643	0.2340	#6 [4.5–6.0] *	1	5.3820	0.2633
	2 ^a^	2.1643	0.2465		2 ^a^	5.1481	0.1688
	3	2.3371	0.2328		3	5.2826	0.1858
	4	2.2473	-		4	5.3174	-

* Interval of allowed lengths for the array elements in the optimization process (in units of $\lambda_{0}$). ^a^ Active element of the antenna.

**Table 3:** Quality factor *Q*, amplitude and phase of $Z_{22}$ and $Z_{in}$, and mutual coupling $\left(Z_{2}^{C}=Z_{in}-Z_{22}\right)$ of the antennas of Table 2. The upper value in each row is related to $\varepsilon_{r}=1.000$, the lower one to $\varepsilon_{r}=1.025$. The quality factor is determined at $\varepsilon_{r}=1.000$.

Antenna Number	Q	$\left|Z_{22}\right|$ (Ω)	$\Phi \left(Z_{22}\right)$ (°)	$\left|Z_{in}\right|$ (Ω)	$\Phi \left(Z_{in}\right)$ (°)	$\left|Z_{2}^{C}\right|$ (Ω)	$\Phi \left(Z_{2}^{C}\right)$ (°)
#1	102.16	311.434	−28.039	50.000	0.000	268.334	146.936
		311.747	−25.392	84.647	−30.663	227.591	156.566
#2	78.41	92.574	−49.308	49.997	−0.001	70.951	98.397
		99.712	−56.485	42.973	−45.174	58.187	115.186
#3	116.98	99.672	−46.001	50.000	0.001	74.235	105.019
		111.222	−57.070	52.560	−58.100	58.679	123.853
#4	225.34	103.189	−42.328	50.000	0.000	74.290	110.723
		117.807	−57.406	80.894	−64.256	38.712	137.026
#5	212.50	107.002	−45.370	49.999	0.000	80.201	108.292
		129.922	−60.104	77.961	−72.933	56.617	137.698
#6	254.33	108.903	−44.823	50.000	0.000	81.458	109.539
		137.228	−60.802	89.242	−74.403	54.676	141.769

**Table 4:** Description of the prototyped antenna at 8.5 GHz. Parameters according to Figure 1A.

Element i	Lengths $l_{i}$ (cm)	Spacing $d_{i}$ (cm)
1	19.0171	0.9528
2 ^a^	18.1553	0.6031
3	18.5847	0.6483
4	18.8143	-

^a^ Active element of the antenna

**Table 5:** Absolute values of S11 of the prototype of the antenna (Figure 9) at the resonant frequency for different times of exposure to PM particles.

Time of Exposure (min)	|S_11_| @ 8.488GHz (dB)
0	−58.49
10	−57.68
20	−56.36
30	−56.12
40	−54.78
50	−53.26
60	−52.76
70	−51.31
80	−50.81
90	−50.48

## Supplementary Materials

The following are available online at https://www.mdpi.com/1424-8220/20/11/3225/s1: Figure S1: Parametric study of the formulation regarding the dielectric constant of the pure air.
