# Supplementary material for: Feasibility Study on Measuring the Particulate Matter Level in the Atmosphere by Means of Yagi–Uda-Like Antennas
Source: Sensors (Basel). 2020 Jun 5;20(11):3225. doi: 10.3390/s20113225 (PMC7308824; doi:10.3390/s20113225)
Supplement: Supplementary file 1 [file sensors-20-03225-s001.pdf]

sensors

MDPI

Article

Feasibility Study on Measuring the Particulate Matter Level in the Atmosphere by Means of Yagi–Uda-Like Antennas

Aarón A. Salas-Sánchez [1,2], Julian Rauch [1], M. Elena López-Martín [3], J. Antonio Rodríguez-González [1], Giorgio Franceschetti [4] and Francisco J. Ares-Pena [1,*]

[1] CRETUS Institute, Department of Applied Physics, University of Santiago de Compostela, E-15782 Santiago de Compostela, Spain; aaronangel.salas@usc.es (A.A.S.S.); rauchju93@googlemail.com (J.R.); ja.rodriguez@usc.es (J.A.R.G.)

[2] ELEDIA@UniTN, Department of Information Engineering and Computer Science, University of Trento, 38123 Trento, Italy;

[3] CRETUS Institute, Department of Morphological Sciences, University of Santiago de Compostela, E-15782 Santiago de Compostela, Spain; melena.lopez.martin@usc.es

[4] Department of Electrical Engineering and Information Technology (DIETI), Università di Napoli Federico II, Naples, 80131 Italy; gfrance@unina.it

* Correspondence: francisco.ares@usc.es

Received: 7 May 2020; Accepted: 2 June 2020; Published: 5 June 2020

Following the mathematical formulation described in (6) we can determine that the dielectric constant obtained by the model for real Earth conditions presents an upper limit of approximately 1.0008.

To demonstrate this statement, several plots of the dielectric constant of the pure air—related with temperature, relative humidity, and pressure of the ambient—are shown. An additional intermediate physical parameter has to be considered within the mathematical description: it is the water vapor pressure (V). This temperature-dependent property of the ambient can be expressed by means of the following semi-empirical formulation [1]:

$$V = e^{A - \frac{B}{T} + CT + D\ln T} \tag{S1}$$

where V is expressed in Pa ($100Pa = 1mbar$) and T is expressed in Kelvin; the constants which apply to this analysis are set [2] as $A = 77.34$, $B = 7235$, $C = 0.005711$, and $D = -8.2$.

Therefore, a set of scenarios is reproduced in Figure S1. These situations illustrate a parametric study of the formulas between –10°C and 40°C with temperature steps of 10°C; atmospheric pressure from 800mbar to 1100mbar; and relative humidity from 0% to 100%.

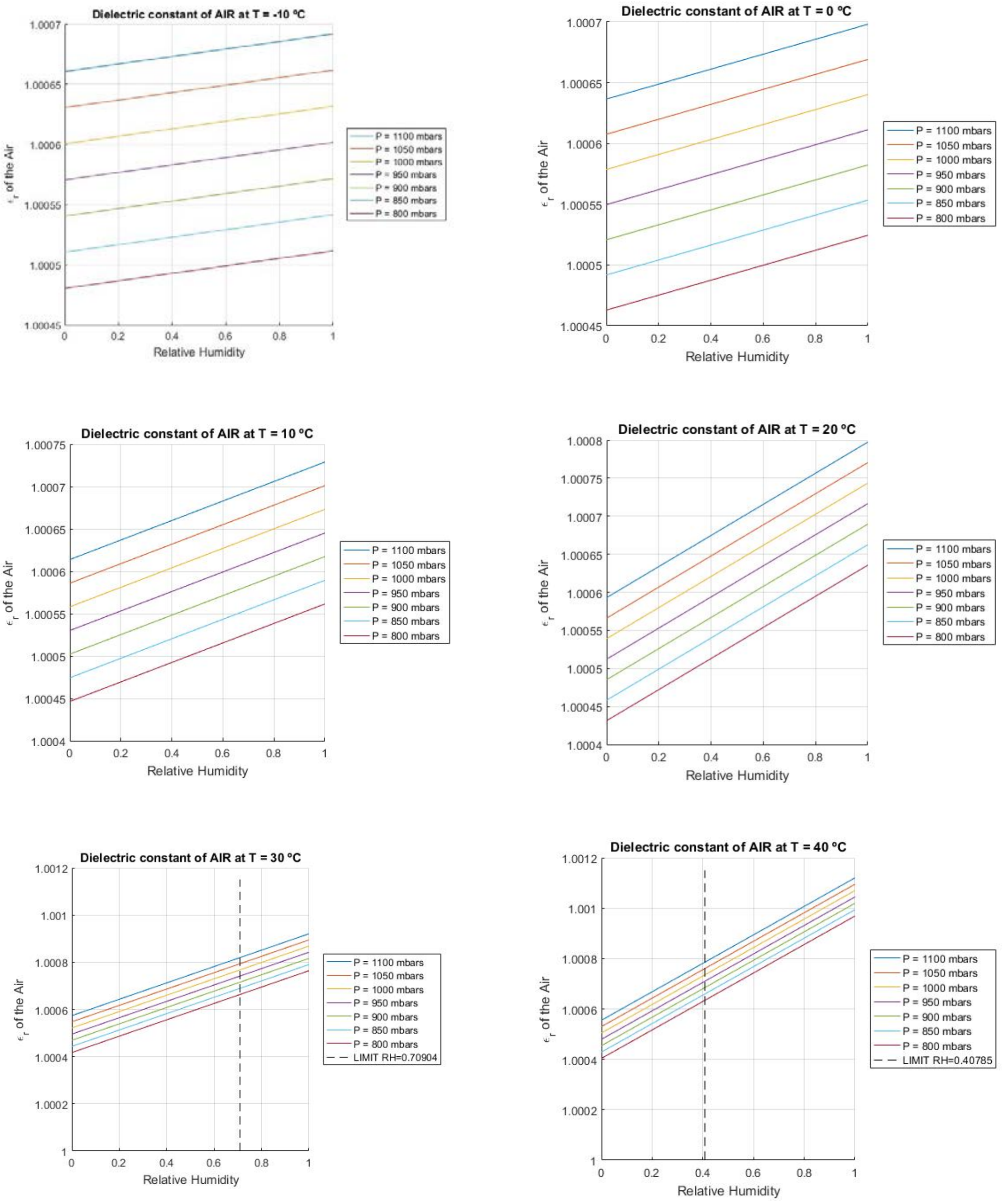

Figure S1. Parametric study of the formulation regarding the dielectric constant of the pure air.

References

1. Zemansky, M.W., Dittman, R.H. *Heat and Thermodynamics*, 6th ed.; McGraw Hill Inc: New York, NY, USA, 1986.
2. Nesmeyanov, A.N. *Vapor Pressure of the Chemical Elements*; Academic Press: New York, NY, USA, 1963.

© 2020 by the authors. Licensee MDPI, Basel, Switzerland. This article is an open access article distributed under the terms and conditions of the Creative Commons Attribution (CC BY) license (http://creativecommons.org/licenses/by/4.0/).
